# Low-Complexity 2D-DOD and 2D-DOA Estimation in Bistatic MIMO Radar Systems: A Reduced-Dimension MUSIC Algorithm Approach

**DOI:** 10.3390/s24092801

**Published:** 2024-04-27

**Authors:** Mushtaq Ahmad, Xiaofei Zhang, Xin Lai, Farman Ali, Xinlei Shi

**Affiliations:** 1 College of Electronic and Information Engineering, Nanjing University of Aeronautics and Astronautics, Nanjing 210016, China; mushtaq@nuaa.edu.cn (M.A.); laixin@nuaa.edu.cn (X.L.); farmanali@nuaa.edu.cn (F.A.); lincoln@nuaa.edu.cn (X.S.); 2 Key Laboratory of Dynamic Cognitive System of Electromagnetic Spectrum Space, Nanjing University of Aeronautics and Astronautics, Ministry of Industry and Information Technology, Nanjing 211106, China

**Keywords:** Bistatic MIMO radar, RD-MUSIC, UPA, 2D-DOD and 2D-DOA estimation, low-complexity algorithm

## Abstract

This paper presents a new technique for estimating the two-dimensional direction of departure (2D-DOD) and direction of arrival (2D-DOA) in bistatic uniform planar array Multiple-Input Multiple-Output (MIMO) radar systems. The method is based on the reduced-dimension (RD) MUSIC algorithm, aiming to achieve improved precision and computational efficiency. Primarily, this pioneering approach efficiently transforms the four-dimensional (4D) estimation problem into two-dimensional (2D) searches, thus reducing the computational complexity typically associated with conventional MUSIC algorithms. Then, exploits the spatial diversity of array response vectors to construct a 4D spatial spectrum function, which is crucial in resolving the complex angular parameters of multiple simultaneous targets. Finally, the objective is to simplify the spatial spectrum to a 2D search within a 4D measurement space to achieve an optimal balance between efficiency and accuracy. Simulation results validate the effectiveness of our proposed algorithm compared to several existing approaches, demonstrating its robustness in accurately estimating 2D-DOD and 2D-DOA across various scenarios. The proposed technique shows significant computational savings and high-resolution estimations and maintains high precision, setting a new benchmark for future explorations in the field.

## 1. Introduction

In bistatic Multiple-Input Multiple-Output (MIMO) radar systems, the estimation of two-dimensional (2D) directions of departure (DOD) and 2D directions of arrival (DOA) is an essential aspect of target localization and parameter estimation. Several research studies have focused on developing algorithms and methods to estimate these parameters accurately [1,2,3,4,5,6]. The challenge of estimating the direction-of-departure (DOD) and direction-of-arrival (DOA) has been thoroughly explored for MIMO radars. However, a limited number of investigations have been done on the issue of estimating 2D-DOD and 2D-DOA, specifically in terms of azimuth and elevation [7,8,9]. Due to its benefits in target detection and parameter estimation, MIMO has attracted considerable interest over the past decade [10,11]. One of the most-studied aspects of MIMO radar signal processing is the estimation of the DOD and DOA [12,13]. Several ways for estimating joint DOA and DOD in bistatic MIMO radar have been proposed [14,15]. Literature [16] suggests a Capon-based estimator; however, it is computationally intensive due to an exhaustive search in a 2D space. The algorithms are based on eigen-subspace theory, of which the Multiple Signal Classification (MUSIC) algorithm [17], the Estimation of Signal Parameters via Rotational Invariance Technique (ESPRIT) algorithm [18], and the Propagator method (PM) [19] are regarded as representatives and have motivated many optimization algorithms. To save computing costs, the PM algorithm does not use eigenvalue decomposition of the signal covariance matrix. However, it performs worse than ESPRIT in low SNR situations. MUSIC algorithm is one of the most widely used eigen-subspace methods, and it has high estimation performance [20]; however, it is harmed by its high computational complexity due to the exhaustive search for the optimal angle directions. The author of [21] developed a reduced-dimension Capon technique that estimates DOD and DOA separately and then requires a pair of matching between the 2D angle estimation. However, This results in an extra processing burden. The RD-PM algorithm [22] simplifies 2D DOA estimation to 1D local search, thus reducing complexity; however, it may suffer accuracy loss and increased errors in noisy environments. The RD-ESPRIT algorithm is proposed in [23] to reduce complexity. Although this comes at the cost of accuracy in more complex scenarios. Aiming to reduce the computational burden further while maintaining accurate DOD and DOA estimation performance, the authors in [24] introduced a reduced dimension MUSIC (RD-MUSIC) algorithm. This approach effectively reduces the complexity from O($n^{2}$) to O(*n*). The MUSIC algorithm and its variant, RD-MUSIC, have been widely used for their easy implementation and high resolution [25]. These algorithms are eigen-subspace-based algorithms that require exact prior information on the effective rank of the covariance matrix. Akaike information criterion (AIC) and minimum description length (MDL) [26,27] are the most frequently used algorithms to estimate the number of sources. They use the eigenvalues of the sample covariance matrix to determine the number of smallest eigenvalues that are expected to be almost equal [26]. However, experimental evidence shows that, for a small sample size and a low signal-to-noise ratio, they tend to estimate a wrong number of sources [28]. It is observed in [26], though AIC criteria perform better than the MDL criteria in low SNR, an estimation error floor is always visible even in the high SNR range, In MDL, higher SNR than in AIC is required to reach error-free estimation. In [29], the authors proposed a MUSIC-Like DOA estimation without estimating the number of the source. These estimation algorithms introduced a new optimization problem under the framework of beamforming rather than in the framework of statistical model identification. The authors in [9], estimated 2D-DOD and 2D-DOA in MIMO radar using the ESPRIT-based EVS component to get closed-form paired azimuth and elevation angles. However, multipath factors cause ambiguity and need extensive computation. In [8], authors use a coprime EMVS to estimate 2D-DOD and DOA for bistatic MIMO radar by arranging covariance data into a computationally complex fourth-order tensor. Additionally, subarray uniformity and coprime configuration affect efficacy. A joint estimate of DOD and DOA in bistatic coprime MIMO radar is suggested in [30], using the technique that relies on enhanced transmit and receive correlation matrices. A modified matrix pencil technique was used in [12] to identify coherent targets, estimate DODs and DOAs, and automate parameter pairing. However, the performance may degrade in noisy environments and be computationally intensive. Authors of [7] construct a difference-coarray by extracting covariance matrix elements and performing angle estimation in sparse bistatic MIMO radar. However, noisy conditions can reduce the accuracy of angle estimations. Consequently, there is a lack of precision and computational efficiency. The vast majority of the aforementioned DOD and DOA estimate algorithms in bistatic MIMO radar suffer from a high computational complexity load, and the noisy environment affects accurate estimation. To tackle these challenges, we propose a joint estimation approach adopting the RD-MUSIC algorithm. This method effectively transforms the four-dimensional (4D) estimation problem into two-dimensional (2D) searches, which leads to a reduction in computational complexity. The proposed technique constructs a 4D spatial spectrum function and then reduces it to a 2D search within a 4D measurement space. The most significant achievement of this method is achieving an optimal balance between efficiency and accuracy.

### Motivation and Contribution

The rapid advancement in MIMO radar systems has attracted considerable interest in enhancing its performance, particularly in the efficient and accurate estimation of directional parameters. The increasing demand for real-time applications underscores the necessity of developing algorithms that are both efficient and have a low computational complexity. Our study offers technological advancements for bistatic MIMO radar systems, as outlined below:

- We present a new RD-MUSIC algorithm for efficient and high-resolution estimation Of 2D-DOD and 2D-DOA in bistatic MIMO radar systems. This innovative approach fundamentally transforms a complex 4D problem into a one-dimensional search. This is a significant advancement for MIMO radar systems.
- We construct a novel 4D spatial spectrum function using the spatial diversity of array response vectors, which is pivotal for accurately resolving the complex angular parameters of multiple targets simultaneously.
- We convert the complex 4D spatial spectrum function into a simplified two-dimensional search within a 4D measurement space. This improves computational efficiency while retaining high accuracy in the estimation process.
- We conduct a comprehensive simulation to demonstrate the exceptional performance of our method. The effectiveness of our algorithm is validated against several state-of-the-art methods, demonstrating considerable computational savings, high-resolution estimation, and robust precision across diverse scenarios.

The paper is structured in the following pattern. Section 2 describes the received signal model in the bistatic MIMO radar with uniform planar array configurations. Section 3 proposes and analyses the proposed algorithm (RD-MUSIC) for joint 2D-DOD and 2D-DOA estimation of MIMO radar. Section 4 presents a comparison of performance analysis, which evaluates the computational complexity of the proposed method. Section 5 includes simulation results to demonstrate the validity of the suggested approach. Finally, Section 5 brings the manuscript to a close.

## 2. MIMO Radar System with Uniform Planar Arrays

**Figure 1:**
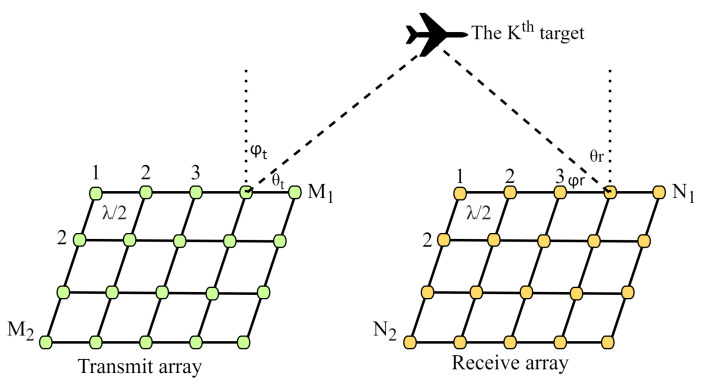
Bistaticic MIMO Radar system UPA configuration.

We consider a MIMO radar system equipped with Uniform Planar Arrays (UPA) for both transmit $T_{R}$ and receive $T_{X}$ arrays. Assume the transmit and receive arrays have *M* and *N* antennas, respectively. The adjacent element spacing is set to λ/2. The schematic of the UPA bistatic MIMO radar system is depicted in Figure 1.

There are *K* uncorrelated targets present in the scenario. The 2D-DOD and 2D-DOA of the $k^{th}$ target, with respect to the transmit and receive arrays, are denoted by $\left(\theta_{tk},\phi_{tk}\right)$ and $\left(\theta_{rk},\phi_{rk}\right)$, respectively. The output of the matched filters at the receiver is expressed as:

(1) $$X\left(t\right)=\left[a_{r}\left(\theta_{r1},\phi_{r1}\right)⊗a_{t}\left(\theta_{t1},\phi_{t1}\right),\ldots ,a_{r}\left(\theta_{rK},\phi_{rK}\right)⊗a_{t}\left(\theta_{tK},\phi_{tK}\right)\right]b\left(t\right)+n\left(t\right).$$

where $\theta_{rk},\phi_{rk}$ are the elevation and azimuth of the 2D-DOA of the $k^{th}$ target, $\theta_{tk},\phi_{tk}$ are the elevation and azimuth of the 2D-DOD of the $k^{th}$ target. The term n(t) is an M×N vector representing Gaussian white noise with zero mean and a covariance matrix $\sigma^{2}I_{MN}$.

The term $b\left(t\right)={\left[b_{1}\left(t\right),b_{2}\left(t\right),\ldots ,b_{K}\left(t\right)\right]}^{T}$ is in $C^{K\times 1}$. The vectors $a_{r}\left(\theta_{rk},\phi_{rk}\right)$ and $a_{t}\left(\theta_{tk},\phi_{tk}\right)$ represent the receive and transmit steering vectors, respectively, for the $k^{th}$ target. The array factor for the receiver is constructed as:

(2) $$a_{r}\left(\theta_{rk},\phi_{rk}\right)=a_{rx}\left(u_{k}\right)⊗a_{ry}\left(v_{k}\right)$$

Here, $a_{rx}\left(u_{k}\right)$ and $a_{ry}\left(v_{k}\right)$ are the receiver array response vectors along x and y dimensions, respectively, where, $a_{rx}\left(u_{k}\right)=\left[1,e^{-j\pi u_{k}},\ldots ,e^{-j\pi \left(N_{1}-1\right)u_{k}}\right]$ and, $a_{ry}\left(v_{k}\right)=\left[1,e^{-j\pi v_{k}},\ldots ,e^{-j\pi \left(N_{2}-1\right)v_{k}}\right]$

(3) $$a_{t}\left(\theta_{tk},\phi_{tk}\right)=a_{tx}\left(u_{k}\right)⊗a_{ty}\left(v_{k}\right)$$

Here, $a_{tx}\left(u_{k}\right)$ and $a_{ty}\left(v_{k}\right)$ are the transmit array response vectors along x and y dimensions, respectively, where, $a_{tx}\left(u_{k}\right)=\left[1,e^{-j\pi u_{k}},\ldots ,e^{-j\pi \left(N_{1}-1\right)u_{k}}\right]$ and, $a_{ty}\left(v_{k}\right)=\left[1,e^{-j\pi v_{k}},\ldots ,e^{-j\pi \left(N_{2}-1\right)v_{k}}\right]$

The spatial frequencies $u_{k}$ and $v_{k}$ are derived from the DOD/DOA angles and are defined as

(4) $$\begin{array}{ccccc} u_{k} & =\frac{2\pi d}{\lambda }\sin\theta_{k}\cos\phi_{k}, & & v_{k} & =\frac{2\pi d}{\lambda }\sin\theta_{k}\sin\phi_{k},\\ u_{k}^{\prime } & =\frac{2\pi d}{\lambda }\sin\theta_{k}^{\prime }\cos\phi_{k}^{\prime }, & \; & v_{k}^{\prime } & =\frac{2\pi d}{\lambda }\sin\theta_{k}^{\prime }\sin\phi_{k}^{\prime }. \end{array}$$

The combined array response for a MIMO radar system using a UPA can be described as follows:

(5) $$A=A_{r}\circ A_{t}$$

Here, $A_{r}=A_{rx}\circ A_{ry},\;\mathrm{and}\;A_{t}=A_{tx}\circ A_{ty}$ are the receive and transmit response matrixes along the *x* and *y* dimensions, respectively. Where, $A_{rx}=\left[a_{rx}\left(u_{1}\right),\ldots ,a_{rx}\left(u_{k}\right)\right],$ $A_{ry}=\left[a_{ry}\left(v_{1}\right),\ldots ,a_{ry}\left(v_{k}\right)\right]$ and $A_{tx}=\left[a_{tx}\left(u_{1}^{\prime }\right),\ldots ,a_{tx}\left(u_{k}^{\prime }\right)\right]$ $A_{ty}=\left[a_{ty}\left(v_{1}^{\prime }\right),\ldots ,a_{ty}\left(v_{k}^{\prime }\right)\right]$. Each of these array response matrices—$A_{rx}$, $A_{ry}$, $A_{tx}$, and $A_{ty}$—is constructed from the array response vectors for each target along their respective dimensions.

The signal vector received by each element of the array antenna is denoted as X

(6) X=AS+N

where A is the steering matrix, S is the signal vector, and N is the noise vector.

Let the covariance matrix for the received signal be denoted as $R_{x}$, which can be estimated with *L* snapshots by,

(7) $$R_{x}=\frac{1}{L}\sum_{l=1}^{L}X\left(t_{l}\right)X^{H}\left(t_{l}\right).$$

Using eigen decomposition, the covariance matrix $R_{x}$ can be decomposed as $R_{x}=E_{s}D_{s}E_{s}^{H}+E_{n}D_{n}E_{n}^{H}$. In this eigen decomposition of $R_{x}$, $D_{s}$ and $D_{n}$ are diagonal matrices containing the largest *K* and the smallest (MN−K) eigenvalues, respectively. $E_{s}$ and $E_{n}$ represent the signal and noise subspaces, respectively, with $E_{n}$ being orthogonal to $E_{s}$. This stage distinguishes signal and noise subspaces.

## 3. 2D-DOD and 2D-DOA Estimation

In this part, we provide a technique for estimating angles in a MIMO radar system using four-dimensional measurements.

### 3.1. Proposed Algorithm

In [24], the authors proposed a variant of the multiple signal classification (MUSIC) algorithm: Reduced-Dimension (RD-) MUSIC algorithm. Since the RD-MUSIC algorithm requires only a one-dimensional search, it saves half of the high computational cost of the traditional MUSIC algorithm. In the RD-MUSIC algorithm, the spatial spectrum function is defined as

(8) $$\begin{array}{c} f=\frac{1}{\left[a\left(u_{k}\right)⊗a\left(v_{k}\right)⊗a\left(u_{k}^{\prime }\right)⊗a\left(v_{k}^{\prime }\right)\right]E_{n}E_{n}^{H}{\left[a\left(u_{k}\right)⊗a\left(v_{k}\right)⊗a\left(u_{k}^{\prime }\right)⊗a\left(v_{k}^{\prime }\right)\right]}^{\prime }} \end{array}$$

The left side of the function is simplified as $a^{H}\left(u_{k},v_{k}\right)\left[a(u_{k},v_{k})⊗I\right]$ where $a\left(u_{k},v_{k}\right)=a\left(u_{k}\right)⊗a\left(v_{k}\right)$, and $a\left(u_{k}^{\prime },v_{k}^{\prime }\right)=a\left(u_{k}\right)⊗a\left(v_{k}\right)$.

(9) $$I=I_{M_{1}}⊗I_{N_{2}}$$

Define $Q=\left[a(u_{k},v_{k})⊗I\right]E_{n}E_{n}^{H}{\left[a(u_{k},v_{k})⊗I\right]}^{H}$

(10) $$f=\frac{1}{a^{H}\left(u_{k}^{\prime },v_{k}^{\prime }\right)Qa\left(u_{k}^{\prime },v_{k}^{\prime }\right)}$$

(11) $$\left(u_{k},v_{k}\right)=\arg\min_{u_{k},v_{k}}\frac{1}{e_{1}^{H}Q{\left(u_{k},v_{k}\right)}^{-1}e_{1}}$$

(12) $$=\arg\max_{u_{k},v_{k}}e_{1}^{H}Q{\left(u_{k},v_{k}\right)}^{-1}e_{1}$$

where $e_{1}a\left(u_{k}^{\prime },v_{k}^{\prime }\right)=1$. $e_{1}={\left[1,0,...,0\right]}^{T}\in R^{M^{\prime }N^{\prime }\times 1}$. The constraint $e_{1}^{H}a_{t}=1$ is to normalize the solutions beyond the trivial solution $a_{t}=0$. When given (u,v), the Lagrangian $L(a_{t},\lambda ,\left(u,v\right))$ is defined as,

(13) $$\begin{array}{ccc} L(a_{t},\lambda ,\left(u,v\right)) & = & a_{t}^{H}Q\left(u,v\right)a_{t}-\lambda \left(e_{1}^{H}a_{t}-1\right) \end{array}$$

where λ is the Lagrange multipler and λ∈R.

According to Slater’s constraint qualification [31], the function of spatial frequencies *u* and *v*

(14) $$\begin{array}{ccccc} h(u,v) & = & g^{+}\left(u,v\right) & = & \frac{1}{e_{1}^{H}Q^{-1}\left(u,v\right)e_{1}} \end{array}$$

(15) $$\begin{array}{c} \left({\hat{u}}_{k},{\hat{v}}_{k}\right)=\arg\min_{\left(\hat{u},\hat{v}\right)\in \left[-\frac{\pi }{2},\frac{\pi }{2}\right]}\frac{1}{e_{1}^{H}Q^{-1}\left(\hat{u},\hat{v}\right)e_{1}} \end{array}$$

It can be solved in a one-dimension exhaustive search method in the range of (u,v) in $\left[-\frac{\pi }{2},\frac{\pi }{2}\right]$. Let the positions of *K* peaks in h(u,v) be denoted as $\left[\left({\hat{u}}_{1}^{\prime },{\hat{v}}_{1}^{\prime }\right),\ldots ,\left({\hat{u}}_{k}^{\prime },{\hat{v}}_{k}^{\prime }\right)\right]$. To estimate the elevation angle ${\hat{\theta }}_{k}$ and the azimuth angle ${\hat{\phi }}_{k}$ of the *k*-th target, we use;

(16) $$\begin{array}{cccc} {\hat{\theta }}_{k} & =\arcsin\left(\sqrt{{\hat{u}}_{k}^{2}+{\hat{v}}_{k}^{2}}\right),\;\;\;\;\;\; & {\hat{\phi }}_{k} & =\arctan\left(\frac{{\hat{v}}_{k}}{{\hat{u}}_{k}}\right) \end{array}$$

For each $\left(u_{k}^{\prime },v_{k}^{\prime }\right)$, k=1,2,…,K, the corresponding transmit vector can be constructed as

(17) $$\begin{array}{cc} {\hat{a}}_{r,k} & =\frac{\hat{\lambda }}{2}Q^{-1}\left(u_{k}^{\prime },v_{k}^{\prime }\right)e_{1}. \end{array}$$

where $\hat{\lambda }=\frac{2}{e_{1}^{H}Q^{-1}\left(\hat{u},\hat{v}\right)e_{1}}$. However, ${\hat{a}}_{r,k}$ has not been paired with ${\hat{a}}_{t,k}$. In [24], the authors proposed to use the least squares (LS) fitting method to pair them. The LS fitting method is equivalent to solving the minimization regression problem

(18) $$\begin{array}{ccc} \min_{c_{k}} & \; & ∥Pc_{k}-g_{k}{∥}_{F}^{2} \end{array}$$

where $g_{k}$ is the angle of every entry in ${\hat{a}}_{t,k}$, i.e., $g_{k}=\mathrm{unwrap}\left(-\mathrm{angle}\left({\hat{a}}_{r,k}\right)\right)$; P is the fitting matrix defined as

$$P=\left[\begin{array}{cc} 1 & 0\\ 1 & 1\\ \vdots & \vdots \\ 1 & M_{1}M_{2}-1 \end{array}\right]$$

The optimal 2D-DOD under the above least square estimator is:

(19) $$\begin{array}{c} \left(\hat{u},\hat{v}\right)=\sin^{-1}\left(c_{k,1}\right) \end{array}$$

where

$$c_{k}=\left[\begin{array}{c} c_{k,0}\\ c_{k,1} \end{array}\right]={\left(P^{⊺}P\right)}^{-1}P^{⊺}g_{k}$$

Then, ${\hat{\theta }}^{\prime },{\hat{\phi }}^{\prime }$ can be constructed accordingly.

(20) $$\begin{array}{cccc} {\hat{\theta }}_{k}^{\prime } & =\arcsin\left(\sqrt{{\hat{u}}_{k}^{\prime 2}+{\hat{v}}_{k}^{\prime 2}}\right), & \; & {\hat{\phi }}_{k}^{\prime }=\arctan\left(\frac{{\hat{v}}_{k}^{\prime }}{{\hat{u}}_{k}^{\prime }}\right) \end{array}$$

Thus far, we have developed a method that simultaneously estimates the 2D-DOD and 2D-DOA estimation for bistatic MIMO radar systems equipped with UPA. This contributes significantly to the efficiency of the algorithm in bistatic MIMO radar systems.

### 3.2. The Main Steps of the Proposed Algorithm

To estimate the 2D-DOD and 2D-DOA for bistatic MIMO radar systems, the following are the primary stages that are included in the method that has been proposed: Algorithm 1 demonstrates an overview of the main steps of the proposed algorithm:
**Algorithm 1** Steps of the proposed algorithm. **Objectives:** Perform joint estimation of 2D-DOD and 2D-DOA for multiple targets in a bistatic MIMO radar system with UPAs using the RD-MUSIC algorithm.
 **Input:** 
$K_{\max},\;\mathrm{snapshots},\;\left\{X_{l}\right\}\;\mathrm{data}\;\mathrm{matrix},\;\mathrm{for}\;l=1,2,\ldots ,L;$
1Calculate and decompose the covariance matrix $R_{x}$ to extract $E_{s}$ and $E_{n}$ as in (7).2Construct the 4D spatial spectrum function f(u,v) using $E_{n}$, and simplify to a 2D search problem (8) and (14).3Conduct a 2D exhaustive search to locate the spectrum peaks, and estimate the 2D-DOD and 2D-DOA angles $\theta_{k}$ and $\phi_{k}$ (15) and (16).4Pair $a_{tx}$ with $a_{rx}$ using LS (18).5Obtain $c_{k}$ and then $\theta_{k}^{\prime }$, $\phi_{k}^{\prime }$ using (19) and (20). **Output:** Optimally estimated 2D-DOD and 2D-DOA angles for each target.

## 4. Performance Analysis

This section analyzes the computational complexity and advantages of the proposed methods.

### 4.1. Computation Complexity Analysis

**Figure 2:**
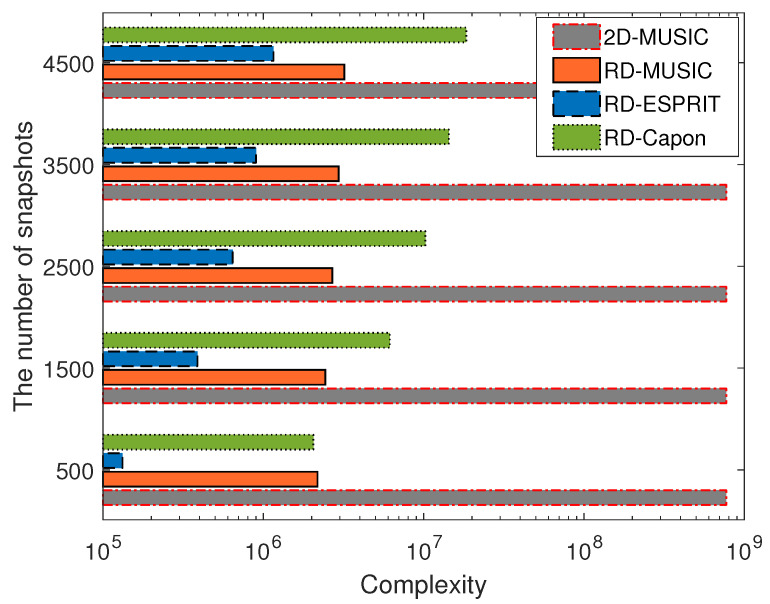
Computational complexity of different algorithms versus the number of snapshots.

We compared the computational complexity of the proposed algorithm with other existing algorithms, including the RD-ESPRIT, RD-Capon, and 2D MUSIC algorithms in Table 1. As depicted in Figure 2, RD-ESPRIT has the lowest computational complexity among the four algorithms, mainly because it does not involve the eigenvalue decomposition of the covariance matrix. It is noticeable that even our technique has a slightly higher computational load than RD-ESPRIT. However, the RD-MUSIC algorithm reduces the complexity of the spatial search and eigenvalue decomposition by projecting the data onto a lower-dimensional subspace. This makes it a computationally more efficient choice for DOA estimation. It also offers better flexibility in dealing with diverse scenarios and parameters in real-world applications and real-time processing capabilities.

### 4.2. Advantages of Proposed Algorithm

Based on the aforementioned analysis, the proposed algorithm presents several significant advantages for bistatic MIMO radar systems, which are summarized as follows:

- The Proposed algorithm significantly decreases the computational complexity by transforming a four-dimensional estimation problem into a two-dimensional search, leading to faster processing times and analysis without sacrificing accuracy, making it highly suitable for real-time radar processing applications.
- By constructing a novel 4D spatial spectrum function, the proposed method achieves high-resolution estimation of 2D-DOD and 2D-DOA, crucial for the precise detection and tracking of multiple targets in bistatic MIMO radar systems.
- The proposed technique outperforms several existing methods across diverse operational scenarios in terms of accuracy and robustness.
- The proposed technique offers significant computational savings, leading to cost reductions in both radar systems’ development and operational phases.

## 5. Simulation Results

The performance evaluations of the proposed method with several existing techniques are covered in this part. RMSE is defined as $\mathrm{RMSE}=\sqrt{\frac{1}{K}\sum_{k=1}^{K}\frac{1}{1000}\sum_{n=1}^{1000}{\left({\hat{\theta }}_{k,n}-\theta_{k}\right)}^{2}}$, where ${\hat{\theta }}_{k,n}$ is the estimated value of the DOD/DOA angle $\theta_{k}$ for the *n*th simulation run.

In the simulation, we present 1000 Monte Carlo simulations to assess the detection performance of the proposed RD-MUSIC algorithm. We normally adopt the bistatic MIMO radar system in the simulations with N=M=8, L=500, $K_{\max}=3$. The three non-coherent sources are all well-separated and located at the angles $\left(\theta_{1},\phi_{1}\right)=\left(10^{\circ }15^{\circ }\right)$, $\left(\theta_{2},\phi_{2}\right)=\left(20^{\circ }25^{\circ }\right)$, $\left(\theta_{3},\phi_{3}\right)=\left(30^{\circ },35^{\circ }\right)$. AWGN channel is assumed. The CRLB = $\mathrm{Var}\left(\hat{\theta }\right)\geq \frac{1}{I(\theta )}$ provides a bound on the variance of an unbiased estimator $\left(\hat{\theta }\right)$ of a deterministic parameter (θ), which supports the RD-MUSIC algorithm’s innovative dimensionality reduction technique.The simulation results provide insights into the algorithm’s performance under various scenarios and parameters, highlighting its accuracy and robustness compared to other existing algorithms.

### 5.1. Spectrum Estimation

**Figure 3:**
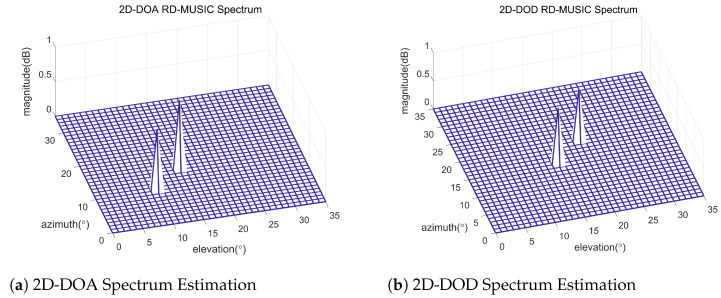
Spectrum estimation of RD-MUSIC algorithm for two different targets, when N=M=8, L=500, $K_{\max}=2$, $\left(\theta_{1},\phi_{1}\right)=\left(10^{\circ },15^{\circ }\right)$, $\left(\theta_{2},\phi_{2}\right)=\left(15^{\circ },20^{\circ }\right)$.

Figure 3 vividly illustrates the results of utilizing our RD-MUSIC method, demonstrating its effectiveness in estimating 2D-DOD and 2D-DOA in a bistatic MIMO radar system that incorporates UPAs based on the assumptions described earlier. The sharp peaks demonstrate the algorithm’s precise detection of the azimuth and elevation angles from which targets arrive. In Figure 3a, the 2D-DOA RD-MUSIC Spectrum is clearly visible in the peaks corresponding to the azimuth and elevation angles for the first target $\left(\theta_{1},\phi_{1}\right)=\left(10^{\circ },15^{\circ }\right)$. This validates the algorithm’s precision in recognizing arrival angles in the multi-dimensional search space. In Figure 3b, the 2D-DOD RD-MUSIC Spectrum depicts the azimuth and elevation angles for the second target’s departure: $\left(\theta_{2},\phi_{2}\right)=\left(15^{\circ },20^{\circ }\right)$. The algorithm’s sensitivity demonstrates its precision in estimating target departure profiles by precisely distinguishing DOD angles. The sharpness of the peaks in the spectrum is directly related to the resolution of the algorithm and more accurate angle estimation. Such precision can potentially improve target estimation in bistatic MIMO radar systems by achieving accuracy with reduced computational complexity.

### 5.2. Scatter Figure

**Figure 4:**
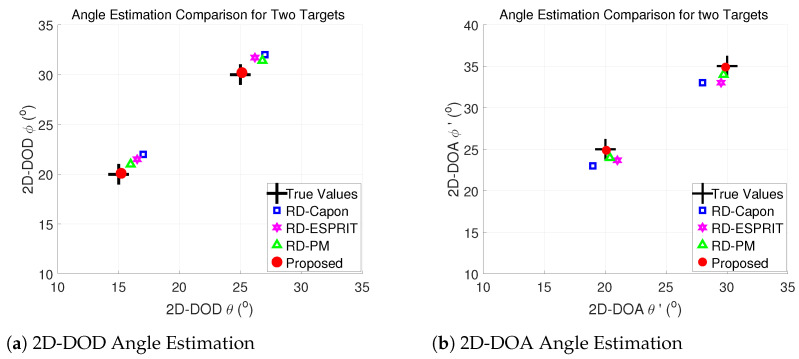
2D angle estimation performance comparison of two different targets.

Here, a graphical representation is generated in Figure 4a,b to visualize the k sources that have been estimated with the proposed and other existing algorithms when M=N=8, SNR=15 dB, L=500, and K=2 at $\left(\theta_{1},\phi_{1}\right)=\left(15^{\circ },20^{\circ }\right)$, $(\theta_{1}^{\prime }$, $\phi_{1}^{\prime })=\left(20^{\circ },25^{\circ }\right)$, $\left(\theta_{2},\phi_{2}\right)=\left(25^{\circ },30^{\circ }\right)$, $\left(\theta_{2}^{\prime },\phi_{2}^{\prime }\right)=\left(30^{\circ },35^{\circ }\right)$, respectively. The cross symbols represent the true values, whereas the solid points depict the estimated values generated by our proposed algorithm. The results in Figure 4 demonstrate that the proposed algorithm exhibits a higher degree of accuracy in estimating all sources.

Table 2 compares estimated target angles using different algorithms. The real values are compared with the estimates obtained from the proposed algorithm, RD-ESPRIT, RD-PM, and RD-Capon. The angular values for each source (ϕ and θ) are shown, highlighting the performance of the algorithms in estimating these values. Table 2 shows that the values estimated by our proposed algorithm are more closely matched to the real values, demonstrating the accuracy of the proposed technique.

### 5.3. Detection Rate vs. SNR

**Figure 5:**
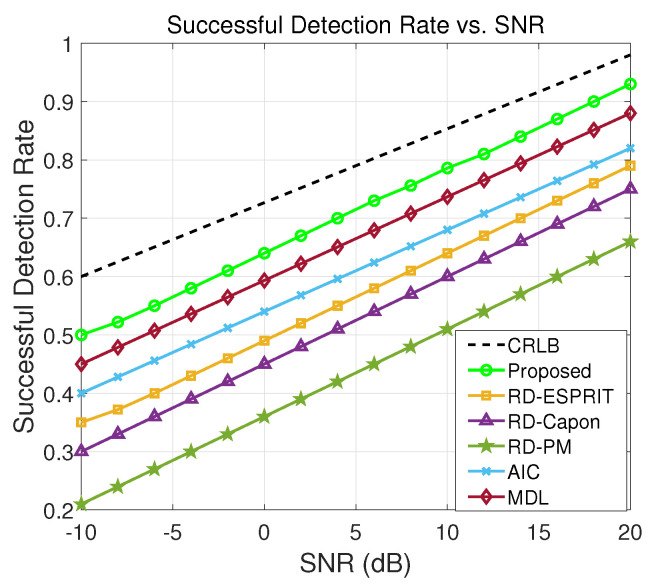
Target detection comparison of different algorithms.

In Figure 5, the successful detection rates of various algorithms are displayed across a range of SNRs. A successful detection rate means how effectively the algorithm detects and locates the presence of targets. Notably, our proposed method consistently exhibits a high successful detection ratio, even in challenging low SNR conditions. This resilience to varying SNR levels underscores the robustness and reliability of our technique in the context of radar system source detection. This is achieved through a combination of dimensionality reduction and the construction of a novel 4D spatial spectrum function, which together enhance the precision of multi-target resolution. Such consistent performance across different SNR regimes positions our approach as a promising and practical choice for real-world applications, where the ability to detect sources accurately and consistently is paramount.

### 5.4. RMSE vs. Snapshot Comparison

**Figure 6:**
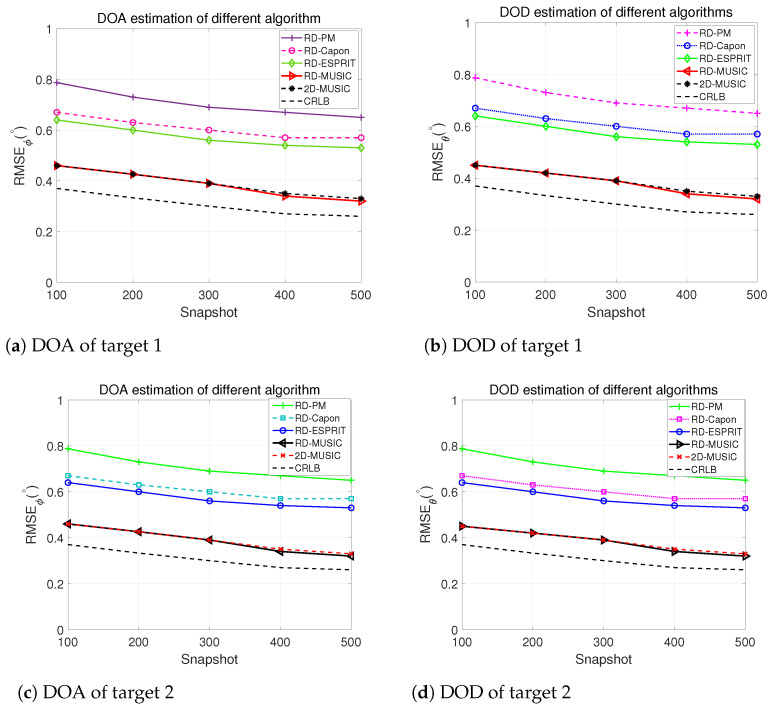
RMSE vs. Snapshot comparison of different algorithms for 2 targets.

Figure 6 demonstrates a comprehensive comparison of our proposed method’s DOA and DOD estimation performance using several existing algorithms, such as RD-ESPRIT, RD-Capon, and RD-PM, at SNR=15 dB. Over 1000 simulations were performed, and the RMSE for each method was calculated with respect to snapshots. Other simulation parameters are the same. Our proposed algorithm showcases remarkable superiority over the alternative algorithms, exhibiting higher accuracy in estimating the angles of arrival and departure while still coming close to the CRLB with a minimal snapshot. Although our proposed algorithm performs similarly to 2D-MUSIC, one-dimensional search supremacy makes our approach advantageous due to complexity reduction.

### 5.5. RMSE vs. SNR Comparison

**Figure 7:**
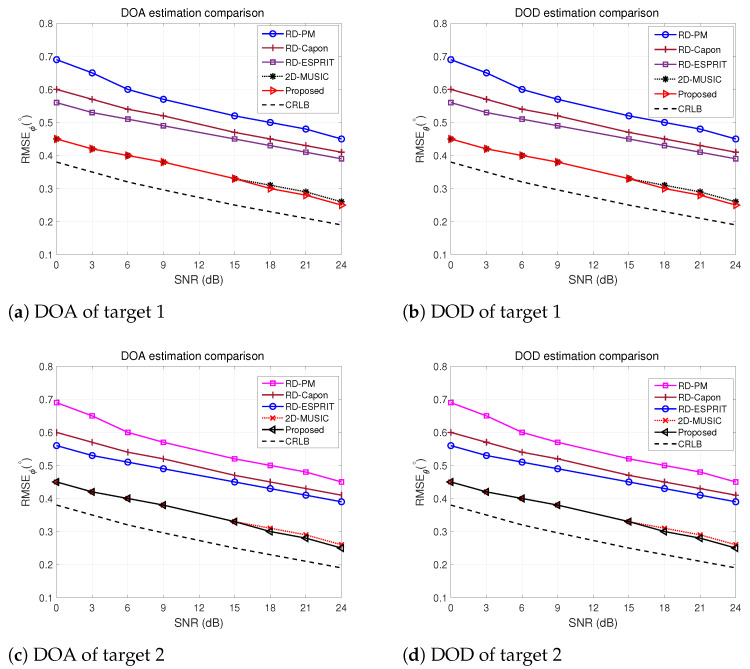
RMSE vs. SNR comparison of different algorithms for 2 targets.

To examine our suggested method further, we evaluated the RMSEs of ϕ and θ versus the SNR, as shown in Figure 7. This assessment is performed for scenarios where the number of snapshots is set to L=500. The figures show that the algorithms’ performance is enhanced with higher SNR values. However, the proposed algorithm offers the lowest RMSEs, close to the CRLB. Our approach apparently outperforms other existing algorithms in angle estimation while closely resembling 2D-MUSIC. However, the proposed algorithm offers considerable computational savings while maintaining exceptional precision, making it more efficient than existing methods.

### 5.6. Performance Comparison with Different L

**Figure 8:**
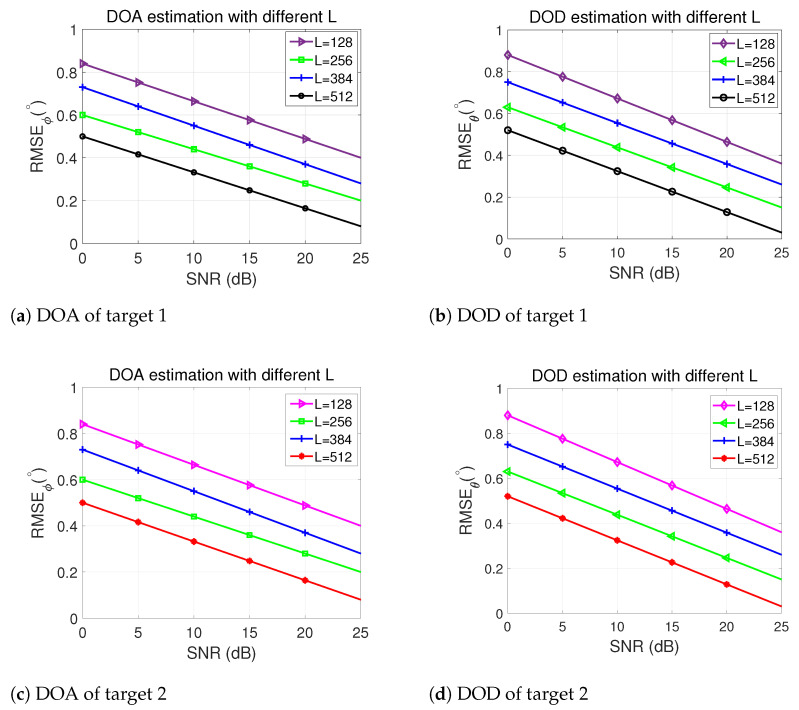
DOA and DOD performance comparison of the proposed algorithm with different L for 2 targets.

Figure 8 demonstrates the angle estimation performance of our proposed algorithm with M = N = 16, SNR = 15 dB, showcasing how this performance varies with different values of L. As observed, an increase in L directly correlates with a noticeable improvement in angle estimation accuracy. The results suggest that as the number of snapshots increases, the algorithm benefits from more accurate covariance estimation by (7), leading to better overall estimation accuracy. Furthermore, the results depicted in Figure 8 demonstrate that the proposed algorithm performs remarkably well even with smaller sampling sizes. This observation showcases the algorithm’s effectiveness across various scenarios, especially in situations where obtaining a large number of snapshots might be challenging or resource-intensive.

## 6. Conclusions

In this paper, a transformative approach has been presented that efficiently addresses the challenges of 2D-DOD and 2D-DOA estimation in bistatic MIMO radar systems with the development of a reduced-dimension MUSIC algorithm. Our method streamlines the complex four-dimensional estimation challenge into a tractable two-dimensional search, thereby significantly enhancing computational efficiency and maintaining high-resolution estimation capabilities. The close alignment with CRLB underscores the proposed algorithm’s ability to achieve near-optimal results. The simulation results validate the proposed algorithm’s capability to outperform existing approaches in terms of both performance and efficiency. Additionally, they demonstrate the algorithm’s practical application in various operating contexts. For future work, we aim to enhance the RD-MUSIC algorithm’s adaptability to diverse operational environments and target dynamics, ensuring robust performance against environmental variations and high target density scenarios.

## Figures and Tables

**Table 1:** Summarizing the computational complexities of the four algorithms (2D-MUSIC, RD-MUSIC, RD-ESPRIT, and RD-Capon).

Algorithm	Complexity Formula
2D-MUSIC	$L\cdot M^{2}\cdot N^{2}+M^{3}\cdot N^{3}+\left(M\cdot N+1\right)\cdot \left(M\cdot N-K\right)\cdot J_{\theta }^{2}$
RD-MUSIC	$L\cdot M^{2}\cdot N^{2}+M^{3}\cdot N^{3}+\left(\left(M^{2}\cdot N+M^{2}\right)\cdot \left(M\cdot N-K\right)+M^{2}\right)\cdot J_{\theta }$
RD-ESPRIT	$L\cdot M^{2}\cdot N^{2}+M^{3}\cdot N^{3}+2\cdot K^{2}\cdot \left(M-1\right)\cdot N+2\cdot K^{2}\cdot \left(N-1\right)\cdot M+6\cdot K^{3}$
RD-Capon	$L\cdot M^{3}\cdot N^{3}+2\cdot M^{2}\cdot N^{2}\cdot \left(M+N-K\right)+4\cdot M\cdot N\cdot \left(M+N\right)+6\cdot M\cdot N\cdot \left(M+N+1\right)$

**Table 2:** Target Estimation Comparison.

Targets	Targets 1				Targets 2			
**Parameters**	$\phi_{1}$	$\theta_{1}$	$\phi_{2}$	$\theta_{2}$	$\phi_{1}^{\prime }$	$\theta_{1}^{\prime }$	$\phi_{2}^{\prime }$	$\theta_{2}^{\prime }$
True values	20	15	30	25	25	20	35	30
Proposed	20.1	15.2	30.2	25.1	24.9	20.1	34.9	29.9
RD-Capon	22	17	32	27	23	19	33	28
RD-ESPRIT	21.5	16.5	31.7	26.2	23.7	21	33	29.5
RD-PM	21	16	31.4	26.8	24	20.1	34	29.9

## Data Availability

Data are contained within the article.
